# A randomized controlled trial to evaluate the effect of incorporating peanuts into an American Diabetes Association meal plan on the nutrient profile of the total diet and cardiometabolic parameters of adults with type 2 diabetes

**DOI:** 10.1186/1475-2891-13-10

**Published:** 2014-01-22

**Authors:** Michelle Wien, Keiji Oda, Joan Sabaté

**Affiliations:** 1 Department of Nutrition, School of Public Health, Loma Linda University, Nichol Hall 1102, Loma Linda, CA 92350, USA; 2 Department of Epidemiology and Biostatistics, Loma Linda University, Loma Linda, CA 92350, USA

**Keywords:** Peanut, Type 2 diabetes, Glucose, Lipids, Weight management

## Abstract

**Background:**

According to the American Diabetes Association (ADA), the nutritional goals for patients with type 2 diabetes (T2D) are to achieve an optimal nutrient intake to achieve normoglycemia and a cardioprotective lipid profile. Peanuts are nutrient dense foods that contain high levels of monounsaturated fat (MUFA) and are a natural source of arginine, fiber, phytosterols, resveritrol, niacin, folate, vitamin E and magnesium, which have the potential for improving blood lipids and glycemic control. This study sought to evaluate the effect of a peanut enriched ADA meal plan on the nutrient profile of the total diet and cardiometabolic parameters in adults with T2D.

**Methods:**

This was a randomized, prospective 24-week parallel-group clinical trial with 60 adults with T2D [age range 34–84 years; body mass index (BMI) range 17.2-48.7 kg/m^2^]. Subjects consumed an ADA meal plan containing ~20% of energy from peanuts (peanut group) or a peanut-free ADA meal plan (control group). Weight, BMI, waist circumference (WC) and nutrient intake from 24-hour recalls were measured every 4 weeks and fasting blood glucose (FBG), HbA1c and blood lipids were measured every 12 weeks. A mixed-model repeated-measures analysis of covariance was performed to assess the significance of changes in the cardiometabolic parameters.

**Results:**

A higher polyunsaturated fat (PUFA) to saturated fat diet ratio and higher intake of MUFA, PUFA, α-tocopherol, niacin and magnesium was observed in the peanut group as compared to the control group (*P* < 0.01-P = 0.04). Both groups experienced mild reductions in weight, BMI, and WC during the study (*P* = 0.01-*P* = 0.03), however there were no differences between the two groups in these measurements or in FBG, HbA1c or blood lipids. For each kilogram of weight loss in the entire cohort there were associations for reductions in WC of 0.48 cm (*P* < 0.01), FBG of 0.11 mmol/l (*P* = 0.01) and HbA1c of 0.07% (*P* < 0.01).

**Conclusions:**

Daily consumption of a peanut enriched (46 g/d) ADA meal plan over 24 weeks improves the nutrient profile of the total diet and is compatible with weight management and improvement in specific blood lipids.

**Trial registration:**

ClinicalTrials.gov NCT00937222

## Background

Type 2 diabetes (T2D) is a chronic disease that involves a heterogeneous group of disorders of intermediary metabolism characterized by glucose intolerance [1]. Medical nutrition therapy (MNT) is an integral component in both the prevention and management of T2D to achieve adequate glycemic control [2]. According to the American Diabetes Association (ADA), the nutritional goals for patients with T2D are to achieve an optimal nutrient intake to achieve normoglycemia and a cardioprotective lipid profile that reduces the risk for cardiovascular disease (CVD) [3]. However, research is lacking on safe and effective nutritional interventions that can enhance the nutrient profile of the total diet and attenuate the elevated glucose levels and dyslipidemia that is commonly observed in adults with T2D.

A meta-analysis of 10 studies involving high monounsaturated fat (MUFA) diets in patients with T2D showed a decrease in fasting blood glucose (FBG) levels, 2-hour post-prandial glucose levels, and all-day blood glucose and blood insulin levels [4]. In addition to glycemic control, maintaining or increasing high density lipoprotein cholesterol (HDL-C) while reducing low density lipoprotein cholesterol (LDL-C) and triglycerides (TG) are key MNT goals in diabetes management. Frequent total nut (peanuts and tree nuts) and peanut butter consumption was associated with a 44% lower risk of incident CVD and a more favorable plasma lipid profile, including lower LDL-C, non-HDL-C, total cholesterol (TC), and apo-B-100 levels among a large cohort of women with T2D [5]. Other studies have shown that the consumption of a high MUFA diet in persons with T2D can achieve a 19-23% reduction in TG and a 4-7% increase in HDL-C levels [6,7].

When substituted for dietary saturated fat (SFA), MUFA may have beneficial metabolic effects in persons with T2D [7]. Peanuts are nutrient dense foods of vegetable origin that contain high levels of MUFA (~25% by weight and ~40% by energy), and they are a natural source of arginine, fiber, phytosterols, resveritrol, niacin, folate, vitamin E and magnesium, which have the potential for improving blood lipids and glycemic control. It is of scientific interest to evaluate different food sources of nutrients for patients with T2D. Thus, the primary aim of this study was to assess the effect of a peanut and/or peanut butter (hereafter referred to as “peanut”) enriched ADA meal plan on changes in the nutrient profile of the total diet, blood lipids, FBG, HbA1c, and anthropometric measurements [weight, BMI and waist circumference (WC)] in adults with T2D.

## Methods

We conducted a randomized prospective parallel-group clinical trial at the Loma Linda University Medical Center Diabetes Treatment Center, Loma Linda, California. The study duration for each participant was 24 weeks. The present study was designed to test the hypothesis that a peanut-enriched ADA meal plan would be more effective than a nut-free ADA meal plan on improving the nutrient profile of the total diet, blood lipids and glycemic control in adults with T2D.

### Eligibility criteria

Adults with a medical diagnosis of T2D for at least 6 months and HbA1c less than 9.0% were recruited through advertisements on the Loma Linda University campus and surrounding communities. Individuals less than 20 years of age, that smoked, had nut allergies or a history of irritable bowel disease or diverticulitis that could be exacerbated by daily peanut intake, were excluded. Potential subjects that were habitual peanut or tree nut consumers must have been willing to discontinue the intake of all peanut and/or tree nuts for 6 weeks prior to their first scheduled clinic visit. Patients with liver disease, renal disease and/or severe dyslipidemia (TG >4.52 mmol/l or TC >7.77 mmol/l) were also excluded. Use of long-acting insulin and statins were permitted if the dose was stable for at least 3 months. Sixty subjects were enrolled and 57 completed the study. This study was approved by the Loma Linda University Institutional Review Board and has therefore been performed in accordance with the ethical standards laid down in the 1964 Declaration of Helsinki and its later amendments. Written informed consent was obtained from all study subjects.

### Study protocol

During the week 0 visit, the daily resting energy expenditure (REE) for each participant was computed using the Harris Benedict equation. For participants with a BMI <25 kg/m^2^ (10% of participants), an individualized ADA meal plan was developed according to the REE results. An activity factor of 1.3 was utilized for all participants. Participants were prescribed a milk-free meal pattern if they stated they were lactose intolerant, and their traditional number of meals and snacks were honored to maximize dietary adherence. For participants with a BMI > 25 kg/m^2^ (90% of participants), the REE for each participant was computed using the Harris-Benedict equation after adjustment of body weight for overweight status, and they were prescribed energy intake deficits of 500 kcal in accordance with the ADA’s guidelines to facilitate modest weight loss [8]. Daily energy levels prescribed ranged between 1000 kcal to 2400 kcal.

Participants were randomly allocated to consume either an ADA meal plan with ~20% of energy from peanuts and to avoid other tree nuts (peanut group), or to consume an ADA meal plan without peanuts and tree nuts (control group). The amount of peanuts was determined based on previously published data reporting favorable changes in glucose and blood lipid levels in subjects with impaired glucose tolerance consuming a diet containing 20% of energy from another MUFA-rich nut [9]. The prescribed ADA meal plans in this study contained 35% total fat (15% MUFA), 45% carbohydrate and 20% protein.

At week 0, each participant met with the study dietitian for a 1-hour counseling session to receive their individualized ADA meal plan. The peanut group participants received dietary instruction on how to select 80% of their remaining energy needs using the ADA Food Exchange System. A supply of commercially available peanuts and/or peanut butter was provided to participants assigned to the peanut group at clinic visits. Participants were allowed to determine if they preferred pre-packaged single-serving peanuts (salted) only, pre-portioned peanut butter (with added salt and oil) only, or a combination of both to maximize dietary adherence. The peanuts were consumed as part of the participant’s customary meals and snacks and were the primary food source of MUFA (40% by energy and 52% total fat by weight) in the peanut group. Due to the 20% energy contribution from the peanuts, control group participants were advised to consume compensatory servings from the meat/meat substitutes (i.e. beef, fish, eggs, cheese, plant-based proteins) and fat (i.e. butter, margarine, mayonnaise, avocado, oil) exchange lists. Both groups were prescribed an equivalent number of carbohydrate (e.g. milk, fruit, bread/cereal) and vegetable exchanges. However, a 1000 kcal ADA meal plan would contain one additional meat/meat substitute exchange and three additional fat exchanges.

### Measurement of nutrient profile of the total diet and cardiometabolic parameters

To ensure dietary adherence and to assess the nutrient profile of the total diet during the study, six unannounced 24-hour recalls (4 weekdays and 2 weekend days) were conducted by phone, one approximately every 4 weeks. In addition, the dietitian reviewed the prescribed number of ADA food exchanges and provided reinforcement at follow-up clinic visits. The six 24-hour recalls were analyzed using Nutrition Data System for Research software version 2006, developed by the Nutrition Coordinating Center, University of Minnesota, Minneapolis, MN, USA.

Cardiometabolic outcomes included weight, BMI, WC, blood lipids (TC, LDL-C, HDL-C, TG), blood lipid ratios (TC:HDL-C, LDL-C:HDL-C), FBG and HbA1c. Height was measured to the nearest centimeter using a stadiometer at week 0. Weight was measured using an internally calibrated segmental body composition scale/analyzer (model TBF-300A, Tanita®, Arlington Heights, IL, USA). BMI was calculated as weight(kg)/height(m^2^). WC was measured to the nearest 0.1 cm, midway between the last rib and the ileac crest.

Venous blood samples were collected at Quest Diagnostics Patient Care Centers after a 12 hour overnight fast at weeks 0, 12 and 24, and all testing was performed at Quest Diagnostics Laboratory, West Hills, CA, USA. Spectrophotometry [10] was used to determine serum glucose, TC, HDL-C, TG and LDL-C (with immunoseparation [11]), and HbA1c was measured using immunoturbidimetry [12].

### Statistical methods

Sample size, power calculations, simple randomization scheme and statistical analysis were performed utilizing SAS version 9.2 (SAS Institute, Cary, NC, USA). The primary outcome measure for performing the power calculation was HDL-C. Using a mean difference of 0.20 mmol/L and SD of 0.24 mmol/L that was obtained from a walnut intervention study conducted in adults with type 2 diabetes, we had 80% power testing at an alpha of 0.05 to detect a difference of at least a 15% change in HDL-C with 46 subjects [13]. Bivariate statistical analysis using the chi-square test for differences in proportions and two-sided independent t-tests were performed on baseline characteristics using a probability value of 0.05.

An intent-to-treat analysis was performed and all percent change values presented are calculated from least-squares means estimated from mixed models. Week 0, 12 and 24 measurements were included in the analysis, with the exception of weight, BMI, and WC that included additional measurements from weeks 4, 8, 16 and 20. For each dependent variable the most appropriate covariance structure was chosen using likelihood ratio tests, and an unstructured, compound symmetry, heterogeneous compound symmetry, autoregressive, or heterogeneous autoregressive covariance structure was applied. The assumption used in the intent-to-treat model with regard to missing data and unmeasured end points for the dropouts was that they were missing at random.

To assess the significance of changes in the anthropometric and metabolic variables, a mixed-model repeated-measures analysis of covariance was used with diet, week, and diet × week interaction as fixed effects, adjusting for baseline measurements of the outcome variable. The mixed model included the treatment effect (control vs. peanut diet: Diet), time effect as a categorical variable (Time), and the interaction term between the two (Diet × Time). Change in weight from baseline (Δ Weight, in kg) was added to the model in light of the influence of weight change on WC and biological measurements. Stratified analysis were conducted for age (≤55y and >55y), gender, baseline BMI (≤30 kg/m^2^ and >30 kg/m^2^) and statin use (taking a statin and no statin use) to assess for potential 3-way interactions with the treatment and time effects. A natural log transformation was performed on outcome variables for the modeling analysis when indicated to improve normality. A histogram and residual plots were used to verify normality after the transformation. The Kenward-Roger method was employed to estimate denominator degrees of freedom for tests of fixed effects and Tukey-Kramer Honestly Significantly Different tests were performed to detect significant pair-wise differences among the two treatments.

## Results

**Figure 1:**
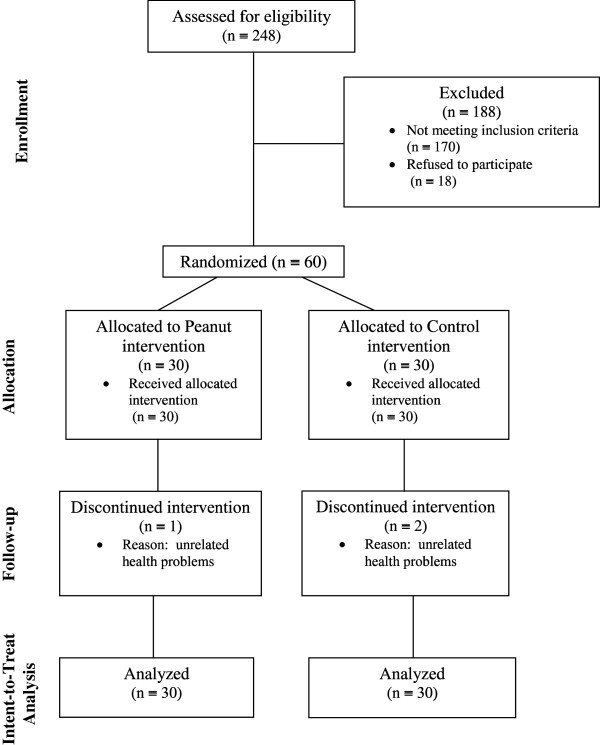
Flow of participants through each stage of the randomized trial.

Sixty adults (age range 34–84 years; BMI range 17.2-48.7 kg/m^2^) met all inclusion criteria and enrolled into the study (peanut group, n = 30; and control group, n = 30) (Figure 1**)**. Three participants withdrew (peanut group, n = 1; and control group, n = 2) during the study due to health problems unrelated to the study.

Participants in both groups were similar in their baseline characteristics **(**Table 1**)** (all *P* > 0.05). Participants in the peanut group consumed either 28 g, 42 g or 56 g of peanuts daily as components of meals and/or snacks to achieve ~20% of their prescribed daily energy level from peanuts, which resulted in a group mean intake of 46 g/d. In the peanut group, 3 participants self-selected to consume peanuts only, 4 participants self-selected to consume peanut butter only, and 23 participants self-selected to consume both peanuts and peanut butter. Participants in the control group met the operational definition of dietary adherence in 97% of the 24-hour recalls, which was to avoid consumption of peanuts, peanut butter and tree nuts. In the peanut group, 83% of the participants consumed between 80-100% of their prescribed dose of peanuts and 17% consumed between 55-79% of their prescribed dose according to the 24-hour recalls. Further, there was no difference in terms of dietary adherence to the prescribed ADA meal plans between the 2 groups.

**Table 1:** Baseline demographic characteristics and biochemical variables by peanut and control groups

	**Peanut (n = 30)**		**Control (n = 30)**	
**Characteristic**	**Mean**	**SD**	**Mean**	**SD**
Age (years)	59	13	64	12
Gender				
Female	13 (57%)		17 (43%)	
Male	17 (43%)		13 (57%)	
Race				
Caucasian	21 (70%)		18 (60%)	
Hispanic	4 (13%)		9 (30%)	
African American	0 (0%)		3 (10%)	
Asian	5 (17%)		0 (0%)	
Duration of diabetes (months)	52.8 (42.9)		72.6 (68.8)	
Antidiabetic treatment				
Diet alone	7 (23%)		4 (13%)	
Metformin alone	12 (40%)		12 (40%)	
Sulfonylurea (SU) alone	1 (3%)		4 (13%)	
Metformin and SU combination	4 (13%)		3 (10%)	
Metformin and thiazolidinedione combination	3 (10%)		2 (7%)	
Other medication combination	3 (10%)		5 (17%)	
Weight (kg)	86.0	24.8	90.4	19.3
BMI (kg/m^2^)	31.1	6.9	33.4	6.8
Waist circumference (cm)	104.3	19.6	110.9	18.5
Plasma lipids, mmol/l				
Total cholesterol	4.39	1.01	4.33	0.78
LDL cholesterol	2.28	0.85	2.23	0.57
HDL cholesterol	1.30	0.49	1.22	0.39
Triglycerides	1.56	0.62	1.92	0.96
Total cholesterol:HDL cholesterol	3.55	1.05	3.74	0.94
LDL cholesterol:HDL cholesterol	1.92	0.87	1.92	0.59
HbA1c (%)	6.6	0.6	6.6	0.6
Glucose, mmol/l	6.27	1.39	6.55	1.22

BMI, body mass index; LDL, low density lipoprotein; HDL, high density lipoprotein.

Values are means ± SD or *n* (%).

Bivariate statistical analysis using the chi-square test for differences in proportions and two-sided independent t-tests were performed on baseline characteristics using a probability value of 0.05. All between group baseline characteristics were *P* > 0.05.

In the context of the United States Food and Drug Administration’s Daily Values that are based on an intake of 2000 kcal/d for adults [14], both groups consumed insufficient amounts of fiber, as well as insufficient amounts of vitamin D, α-tocopherol, calcium, magnesium and potassium. In light of the predominance of hypocaloric ADA meal patterns, the mean self-reported energy intake for both groups was only ~1600 kcal/d, but the MUFA, polyunsaturated fat (PUFA), α-tocopherol, niacin, and magnesium was 25%, 31%, 30%, 18% and 23% higher in the peanut group as compared to the control group, respectively (*P* < 0.01-*P* = 0.04) **(**Table 2**)**. A 40% higher PUFA:SFA (P:S) ratio was also observed in the peanut group (*P* < 0.01). Compared to the peanut group, the mean intake of copper was 5% higher in the control group (*P* < 0.01).

**Table 2:** Nutrient profile of the total diet for the peanut and control groups from the six 24-hour recalls during the study

	**Peanut (n = 29)**		**Control (n = 28)**		***P *** **value**^**a**^
**Nutrient**	**Mean**	**SD**	**Mean**	**SD**	
Energy, kcal	1599	397	1598	443	0.99
Total carbohydrate, g	174	43	189	56	0.28
Total protein, g	74	19	71	20	0.53
Total fat, g	72	26	65	24	0.29
SFA, g	20	8	22	9	0.26
MUFA, g	30	11	24	10	0.04
PUFA, g	17	6	13	5	<0.01
PUFA:SFA (P:S) ratio	1.01	0.27	0.72	0.27	<0.01
Cholesterol, mg	210	143	270	136	0.11
Fiber, g	22	6	19	7	0.19
Vitamin C, mg	83	46	71	33	0.28
Vitamin D, μg	3.4	2.3	3.6	1.6	0.81
α-tocopherol equivalents, mg	11.8	4.3	9.1	4.4	0.02
Niacin, mg	23.5	6.5	20.0	5.9	0.03
Folate, μg	402	119	385	122	0.58
Vitamin B12, μg	5.6	7.4	4.3	1.7	0.36
Calcium, mg	703	292	763	286	0.42
Phosphorus, mg	1114	263	1092	303	0.77
Magnesium, mg	302	53	245	68	<0.01
Copper, mg	9.8	2.4	10.3	3.6	<0.01
Sodium, mg	2703	817	2973	855	0.22
Potassium, mg	2557	550	2298	647	0.10

^a^*P* values are for between-group differences in mean nutrient profile of the total diet.

The intent-to-treat analyses results on the anthropometric and metabolic measurements are presented based on the absolute change in LSM **(**Table 3**)**. Both groups experienced declines in weight (*P* = 0.03), BMI (*P* = 0.02) and WC (*P* = 0.01) during the study, however there were no significant differences in these measurements between the two groups at any time point (Diet × Time interaction *P* = 0.43-0.95). More specifically, the peanut group showed a mean reduction in weight of 0.83 kg as compared to 0.76 kg in the control group. For each kilogram of weight loss in the entire cohort, there were associations for reductions in WC of 0.48 cm (*P* < 0.01), FBG of 0.11 mmol/l (*P* = 0.01) and HbA1c of 0.07% (*P* < 0.01) **(**Table 4**)**.

**Table 3:** **Anthropometric and metabolic parameters for the peanut and control groups at week 24**^**a**^

			**Absolute change & (%) from baseline**			
	**Peanut**	**Control**	**Peanut**	**Control**	***P *** **value**^**c **^**within group**	***P *** **value**^**d **^**between group**
**Parameter**	**LSM**^**b **^**[95%CI]**	**LSM [95%CI]**				
Weight^e^ (kg)	85.2 [77.6, 92.8]	89.7 [81.9, 97.4]	−0.83 (−0.96)	−0.76 (−0.84)	0.03	0.42
BMI^f^ (kg/m^2^)	30.8 [28.4, 33.2]	33.1 [30.7, 35.6]	−0.26 (−0.84)	−0.25 (−0.75)	0.02	0.19
Waist circumference^g^ (cm)	102.6 [95.9, 109.4]	109.8 [103.0, 116.5]	−1.64 (−1.57)	−1.13 (−1.02)	0.01	0.14
Total cholesterol (TC)^h^ (mmol/l)	4.27 [3.94, 4.61]	4.22 [3.88, 4.53]	−1 (−0.57)	−5 (−2.73)	0.52	0.79
LDL cholesterol^h^ (mmol/l)	2.20 [1.94, 2.49]	2.15 [1.86, 2.43]	−3 (−3.30)	−2 (−2.97)	0.72	0.76
HDL cholesterol^h,i^ (mmol/l)	1.30 [1.16, 1.45]	1.22 [1.09, 1.35]	+2 (+5.13)	+1 (+3.05)	0.06	0.45
Triglycerides^h,i^ (mmol/l)	1.36 [1.12, 1.64]	1.54 [1.27, 1.85]	−6 (−4.88)	16 (−10.42)	0.06	0.39
TC:HDL-C^g^	3.38 [2.97, 3.78]	3.56 [3.15, 3.96]	−0.17 (−4.79)	−0.18 (−4.81)	0.08	0.53
LDL-C:HDL-C^g^	1.77 [1.47, 2.07]	1.83 [1.53, 2.14]	−0.15 (−7.63)	−0.09 (−4.63)	0.07	0.77
HbA1c^g^ (%)	6.71 [6.43, 6.99]	6.53 [6.25, 6.81]	+0.12 (+1.82)	−0.10 (−1.51)	0.79	0.38
Glucose^j^ (mmol/l)	6.49 [5.83, 7.01]	6.16 [5.49, 6.77]	+4 (+3.43)	−7 (−6.78)	0.33	0.48

BMI, body mass index; LDL, low density lipoprotein; HDL, high density lipoprotein.

^a^All outcome variables in this intent-to-treat analysis were adjusted for their baseline values in the models, and all baseline values were estimated from the model.

^b^Least squares mean, LSM.

^c^Within group differences from baseline to week 24.

^d^Between group differences at week 24.

^e^*p* values for treatment differences for percent change are based on mixed models with a heterogeneous autoregressive covariance structure with all time points included in the model.

^f^*p* values for treatment differences for percent change are based on mixed models with an autoregressive covariance structure with all time points included in the model.

^g^*p* values for treatment differences for percent change are based on mixed models with an unstructured covariance with all time points included in the model.

^h^*p* values for treatment differences for percent change are based on mixed models with a compound symmetric covariance structure with all time points included in the model.

^i^LSMs are based on log transformation values for the modeling analysis.

**Table 4:** Influence of weight loss on waist circumference, fasting glucose and HbA1c in the entire study cohort

	**Weight loss (−1 kg)**^**a**^	***P *** **value**
Waist circumference (cm)	−0.48	<0.01
Fasting glucose (mmol/l)	−0.11	0.01
HbA1c (%)	−0.07	<0.01

^a^According to regression coefficients in the mixed model analysis.

Seventeen participants (57%) in the peanut group and 15 (50%) in the control group were taking lipid-lowering medications. There was no significant change in TC and LDL-C within or between the groups. Borderline significant increases in HDL-C (+5% peanut group, +3% control group) and decreases in TG (−5% peanut group, -10% control group) were found within the two groups (both *P* = 0.06), which yielded borderline significant reductions in TC:HDL-C (both −5%) (*P* = 0.08) and LDL-C:HDL-C (−8% peanut group, -5% control group) (*P* = 0.07). Lastly, no 3-way interactions were observed in the blood lipids and lipid ratios when stratified by age (≤55y or >55y), gender, BMI (≤30 kg/m^2^ or >30 kg/m^2^) or statin use (taking a statin or no statin use).

During the study, 1 subject in each group had an increase in a medication dose and 1 subject in each group had a decrease in a medication dose according to the recommendations of their respective physicians. The peanut group experienced an absolute increase of 0.12% in HbA1c compared to an absolute decrease of 0.10% in the control group (*P* = 0.38).

## Discussion

This randomized trial in free-living adults with T2D showed that the incorporation of approximately 46 g/d of peanuts into an ADA meal plan yielded a higher P:S ratio and higher intake of MUFA, PUFA, α-tocopherol, niacin and magnesium as compared to a peanut-free control diet, which are important cardioprotective nutrients for persons with T2D. As expected, the peanut intervention yielded similar reductions in weight, BMI and WC as compared to the peanut-free intervention, and all of the blood lipids and lipid ratios improved in a favorable direction in both groups during the study.

Similar to the blood lipid findings of our recent pooled analysis of 25 nut (walnut, almond, macadamia, pistachio, hazelnut, pecan and peanut) intervention trials (583 men and women with normolipidemia and hypercholesterolemia who were not taking lipid-lowering medications) [15], age and gender did not modify the effect of peanuts on the blood lipids and lipid ratios. Contrary to the findings of the aforementioned pooled analysis, BMI did not influence the blood lipid results, perhaps due to the narrower range of BMIs, smaller sample size, and the presence of T2D among the participants in the current study.

The clinically relevant reduction in LDL-C:HDL-C observed in the peanut group and the increase in HDL-C are worth noting in the context of our primarily overweight or obese study population. We have previously cited several reasons for the decreased blood lipid responsiveness to nut enriched diets in overweight and obese individuals [16]. Specifically, obesity is associated with reduced intestinal cholesterol absorption [17]; hence the cholesterol lowering effects from the plant sterols in nuts will be blunted when cholesterol absorption rates are low. Insulin resistant states, the hallmark of overweight and obese persons with T2D, increase cholesterol synthesis and also reduce intestinal absorption [18]. Therefore, enhanced cholesterol flux in hepatocytes down-regulates LDL-C receptors and makes them refractory to changes in dietary fatty acids, and a decreased cholesterol flux through enterocytes reduces the cholesterol-raising response to dietary cholesterol and enhances the aforementioned cholesterol-lowering effect of plant stanols.

Several studies have been performed to determine the influence between specific nuts and blood lipids and glycemic control in persons with T2D. Scott et al. [19] compared an American Heart Association diet [30% fat (15% MUFA), 55% carbohydrate, 15% protein] with an almond enriched high protein diet [40% fat (22% MUFA), 35% carbohydrate, 25% protein] in 7 patients with T2D (unknown degree of lipid lowering medication usage) and observed no between-treatment effects on LDL-C, TG and FBG, which is consistent with our null findings for these measurements. However, these investigators reported that weight loss was a potential confounding factor in the analysis. As a known confounder for influencing WC and biological measurements, the inclusion of weight change was added into our mixed models. As expected, each kilogram of weight loss was associated with significant reductions in WC, FBG and HbA1c among the cohort of study participants (*P* < 0.01-*P* = 0.01) (Table 4), which demonstrates that regardless of either dietary approach the key to improvements in these measurements is mediated through successful weight loss.

More recently, Jenkins et al. [20] evaluated the effects of a 3 month dietary intervention using a parallel study design in 117 subjects with T2D using a full dose of mixed nuts (mean intake 73 g/d), muffins, or half portions of muffins and mixed nuts, hence replacing mixed nuts for carbohydrates in the ADA diet. In contrast to our null findings, this team reported an absolute −0.21% reduction in HbA1c (*P* = 0.001), a 5% decrease in TC (*P* < 0.001), a 8% decrease in LDL-C (*P* < 0.001), a 8% decrease in TC:HDL-C (*P* = 0.006), and a 9% decrease in LDL-C:HDL-C (*P* = 0.002) in the full dose nut group (n = 40) as compared to the muffin group (n = 39).

Our study is not without limitations. The nutrient intake data was obtained from self-reported 24-hour dietary recalls and we did not have a biomarker of dietary adherence. Hence, self-reporting errors in the treatment diet may partially explain the dissociation between the superior nutrient profile of the peanut diet and the lack of difference in cardiovascular disease risk factors. Additionally, we did not obtain 2-hour postprandial blood glucose measurements or utilize a continuous glucose monitoring system to assess the degree of blood glucose variability in the study participants, the latter being associated with oxidative stress and endothelial dysfunction independent of HbA1c [21].

## Conclusions

This study indicates that the daily inclusion of 46 g of peanuts and/or peanut butter (as recommended by the 2003 Food and Drug Administration qualified health claim for nuts) [22] in free-living adults with T2D results in an enhancement of the nutrient profile of the total diet with cardioprotective properties (higher MUFA, PUFA, P:S ratio, α-tocopherol, niacin, and magnesium). Additionally, it also provides a reduction in body weight, BMI, WC and improvement in specific blood lipids and lipid ratios in the context of meal plans developed to produce gradual weight loss; however, not different than the peanut-free ADA meal plan. Therefore, regardless of either dietary approach, the key to improvements in WC, FBG and HbA1c are mediated through successful weight loss. Although we failed to find a difference in the anthropometric measurements between the 2 groups, peanuts and peanut butter were not observed to be obesogenic. Hence, they are convenient, viable and palatable food options that could be easily incorporated into ADA meal plans prescribed to T2D patients, and they are compatible with weight management and improvement in specific blood lipids.

## Abbreviations

ADA: American Diabetes Association; BMI: Body mass index; CVD: Cardiovascular disease; FBG: Fasting blood glucose; HDL-C: High density lipoprotein cholesterol; LDL-C: Low density lipoprotein cholesterol; LSM: Least squares mean; MNT: Medical nutrition therapy; MUFA: Monounsaturated fatty acid; PUFA: Polyunsaturated fat; REE: Resting energy expenditure; SFA: Saturated fat; T2D: Type 2 diabetes; TC: Total cholesterol; TG: Triglycerides; WC: Waist circumference.

## Competing interests

This study was funded by a grant from the National Peanut Board.

## Authors’ contributions

MW and JS designed and coordinated the study. MW was responsible for data collection, analysis and quality control. MW, JS and KO were involved in the statistical analyses. All authors contributed to the interpretation of data. MW wrote the first draft of the manuscript and all authors critically reviewed and revised the manuscript. JS obtained the funding for the study. The authors have no conflicts of interest or competing interests. All authors have given approval of the final version of the manuscript.
